# Tobacco, alcohol, cannabis, and illicit drug use and their association with CD4/CD8 cell count ratio in people with controlled HIV: a cross-sectional study (ANRS CO3 AQUIVIH-NA-QuAliV)

**DOI:** 10.1186/s12879-022-07963-6

**Published:** 2023-01-09

**Authors:** Sophie Devos, Fabrice Bonnet, Mojgan Hessamfar, Didier Neau, Marc-Olivier Vareil, Olivier Leleux, Charles Cazanave, Nicolas Rouanes, Pierre Duffau, Estibaliz Lazaro, François Dabis, Linda Wittkop, Diana Barger, ANRS CO3-AQUIVIH-NA-QuAliV, P. Bellecave, P. Blanco, S. Bouchet, D. Breilh, S. Desjardin, V. Gaborieau, A. Gimbert, L. Lacaze-Buzy, D. Lacoste, M. E. Lafon, S. Lawson-Ayayi, F. Le Marec, G. Le Moal, D. Malvy, L. Marchand, P. Mercié, I. Pellegrin, A. Perrier, V. Petrov-Sanchez, N. Bernard, D. Bronnimann, H. Chaussade, D. Dondia, I. Faure, P. Morlat, E. Mériglier, F. Paccalin, E. Riebero, C. Rivoisy, M. A. Vandenhende, L. Barthod, F. A. Dauchy, A. Desclaux, M. Ducours, H. Dutronc, A. Duvignaud, J. Leitao, M. Lescure, D. Nguyen, T. Pistone, M. Puges, G. Wirth, C. Courtault, F. Camou, C. Greib, J. L. Pellegrin, E. Rivière, J. F. Viallard, Y. Imbert, M. Thierry-Mieg, P. Rispal, O. Caubet, H. Ferrand, S. Tchamgoué, S. Farbos, H. Wille, K. Andre, L. Caunegre, Y. Gerard, F. Osorio-Perez, I. Chossat, G. Iles, M. Labasse-Depis, F. Lacassin, A. Barret, B. Castan, J. Koffi, A. Saunier, J. B. Zabbe, G. Dumondin, G. Beraud, M. Catroux, M. Garcia, V. Giraud, JP. Martellosio, F. Roblot, T. Pasdeloup, A. Riché, M. Grosset, S. Males, C. Ngo Bell, C. Carpentier, C. Tumiotto, G. Miremeont-Salamé, D. Arma, G. Arnou, M. J. Blaizeau, P. Camps, M. Decoin, S. Delveaux, F. Diarra, L. Gabrea, W. H. Lai, E. Lenaud, D. Plainchamps, A. Pougetoux, B. Uwamaliya, K. Zara, V. Conte, M. Gapillout

**Affiliations:** 1 Univ. Bordeaux, INSERM, BPH, U1219, 146, rue Léo Saignat-CS61292, 33076 Bordeaux Cedex, France; 2 CHU de Bordeaux, COREVIH Nouvelle Aquitaine, INSERM, U1219, 1 Rue Jean Burguet, 33000 Bordeaux, France; 3 CHU de Bordeaux, Service de Médecine Interne et Maladies Infectieuses, INSERM, U1219, 1 Rue Jean Burguet, 33000 Bordeaux, France; 4 Univ. Bordeaux, INSERM, Institut Bergonié, BPH, U1219, CIC-P 1401, 146, rue Léo Saignat-CS61292, 33076 Bordeaux Cedex, France; 5 CHU de Bordeaux, Service des Maladies Infectieuses et Tropicales, INSERM, U1219, Pl. Amélie Raba Léon, U121933000 Bordeaux, France; 6 Centre Hospitalier de la Côte Basque, Service de Maladies Infectieuses, 13 Avenue de l’interne Jacques Loëb, BP 8, 64109 Bayonne Cedex, France; 7 Centre Hospitalier de Périgueux, Service de Médecine Polyvalente, 80 Av. Georges Pompidou, 22400 Périgueux, France; 8 Univ. Bordeaux, Department of Immunology, CNRS, ImmunoConcEpT, UMR 5164, 33000 Bordeaux, France; 9 CHU de Bordeaux, Service de Médecine Interne, 1 Avenue de Magellan, 33600 Pessac, France; 10 Univ. Bordeaux, INSERM, INRIA, BPH, U1219, 146, rue Léo Saignat-CS61292, 33076 Bordeaux Cedex, France; 11 CHU de Bordeaux, Service d’information médicale, INSERM, U1219, 146, rue Léo Saignat-CS61292, 33076 Bordeaux Cedex, France

**Keywords:** CD4/CD8 ratio, Chronic inflammation, Chemsex, Drug use, HIV

## Abstract

**Background:**

To evaluate drug use (alcohol, tobacco, cannabis and other drugs) and its association with mean CD4/CD8 T cell count ratio, a marker of chronic inflammation, in virally suppressed people living with HIV-1 (PLWH) in Nouvelle Aquitaine, France.

**Methods:**

A multi-centric, cross-sectional analysis was conducted in 2018–19 in the QuAliV study—ANRS CO3 AQUIVIH-NA cohort. Tobacco, alcohol, cannabis, and other drug use (poppers, cocaine, amphetamines, synthetic cathinones, GHB/GBL) were self-reported. CD4 and CD8 T cell counts and viral load measures, ± 2 years of self-report, and other characteristics were abstracted from medical records. Univariable and multivariable linear regression models, adjusted for age, sex, HIV risk group, time since HIV diagnosis, and other drug use were fit for each drug and most recent CD4/CD8 ratio.

**Results:**

660 PLWH, aged 54.7 ± 11.2, were included. 47.7% [315/660] had a CD4/CD8 ratio of < 1. Their mean CD4/CD8 ratio was 1.1 ± 0.6. 35% smoked; ~ 40% were considered to be hazardous drinkers or have alcohol use disorder; 19.9% used cannabis and 11.9% other drugs. Chemsex-associated drug users’ CD4/CD8 ratio was on average 0.226 (95% confidence interval [95% CI] − 0.383, − 0.070) lower than that of non-users in univariable analysis (p = 0.005) and 0.165 lower [95% CI − 0.343, 0.012] in multivariable analysis (p = 0.068).

**Conclusions:**

Mean differences in CD4/CD8 ratio were not significantly different in tobacco, alcohol and cannabis users compared to non-users. However, Chemsex-associated drug users may represent a population at risk of chronic inflammation, the specific determinants of which merit further investigation.

*Trial registration number:* NCT03296202.

## Background

Residual immune activation and persistent inflammation have now become hallmarks of Human Immunodeficiency Virus (HIV) infection [1, 2]. A low or inverted (< 1) CD4 to CD8 T cell count ratio (CD4/CD8 ratio) is a recognized indirect marker of this impaired immune function [3, 4] and only one-third of people living with HIV (PLWH) see a “normalization” of their CD4/CD8 ratio despite adhering to antiretroviral therapy (ART) and achieving sustained viral suppression [5, 6]. A low CD4/CD8 ratio has been associated with a higher risk of common co-morbidities (cardiovascular, renal and hepatic diseases as well as cancers) and mortality [7–9]. Gaining a better understanding of the mechanisms responsible for immune activation remains an ongoing priority.

Smoking, alcohol misuse and other drug use (i.e. cocaine and cannabis), which are common in PLWH, appear to affect the immune system, specifically its lymphocytes, already targeted by HIV infection [10–20]. Furthermore, “Chemsex”, the phenomenon of drug use to enhance sexual pleasure, has become increasingly common in men who have sex with men (MSM), a population also living with HIV, with Poppers, GHB or GBL, cocaine, amphetamines, and more recently synthetic cathinones being the drugs most frequently taken [21]. We hypothesized that a low CD4/CD8 ratio could be a mediator of the relationship between drug use and the occurrence of common co-morbidities. We aimed to assess the relationship between reported drug use (alcohol, tobacco, cannabis and other drugs) and the CD4/CD8 ratio in virally suppressed PLWH.

## Methods

### Setting, study design, population

The ANRS CO3 AQUIVIH-NA cohort is an open, prospective and multi-centric cohort of PLWH in hospital-based HIV care in 15 public hospitals in the Nouvelle Aquitaine region of France. Clinical and epidemiological data, reflecting routine care from patients’ medical records are entered into an electronic Case Report Form by Clinical Research Associates. Participants’ laboratory reports are transferred from the hospitals’ laboratory information systems. The QuAliV study is a cross-sectional study, conducted within the ANRS CO3 AQUIVIH-NA cohort, assessing the multi-dimensional quality of life and other patient-reported outcomes (PROs) in PLWH via a self-administered assessment [22, 23]. Cohort participants were recruited to the QuAliV study during their routine HIV consultation and were are invited to complete the self-assessment independently via patient-facing module of the cohort’s information system designed for the collection of electronic PROs. We conducted an analytical cross-sectional study to assess the association between drug use and the most recent CD4/CD8 ratio among virally-suppressed PLWH in care in one of six centres recruiting participants to the QuAliV study between 07/2018 and 12/2019. To be included in this analysis, participants had to have completed the “My lifestyle” module of PROs assessment which details tobacco, alcohol, cannabis and other drug use and have available CD4 and CD8 cell count measures within ± 2 years of the self-assessment.

### Outcome

Clinical guidelines for PLWH on treatment with an undetectable viral load recommend routine blood monitoring, including CD4 and CD8 cell counts, at least every two years [13]. We therefore considered CD4 and CD8 T cell counts, measured on the same day, to calculate the CD4/CD8 ratio closest to the date of self-report. As there is no clear threshold for a “normal” CD4/CD8 ratio, we treated the CD4/CD8 ratio as a continuous variable.

### Exposures

Tobacco, alcohol, cannabis, and drug use (poppers, cocaine, amphetamines/ecstasy/MDMA/methamphetamines, opiates, synthetic cathinone, GHB/GBL or synthetic cannabinoids) were self-reported, collected through valid, widely-used, instruments (Fagerström Test for Nicotine Dependence, Alcohol Use Disorders Identification Test (AUDIT-C)) [22]. Participants reported the frequency and location of use (e.g. private home, clubs, pubs, restaurant, street, parks, etc.). We created a variable for current tobacco use based on responses to a single question about smoking and responses to the Fagerström Test for Nicotine Dependence, a 6-item instrument designed to assess the intensity of physical addiction to nicotine which generates a score of 0–10. We classified participants as non-smoking, non-daily smoking or very low to low addiction to nicotine (score 0–4), medium addiction to nicotine (score 5–6) and strong to very strong addiction to nicotine (score 7–10). We created a variable for alcohol use using data generated from the AUDIT-C instrument, a 3-item questionnaire designed to screen for hazardous drinking or those who have active alcohol use disorders. We classified participants as non-drinkers, no misuse, or hazardous drinkers/alcohol use disorder. Cannabis use in the past 12 months was dichotomized as non-user versus user. Other drug use in the past 12 months was collected via a single question on use of "other drugs to feel better or get high". We created a composite variable identifying those who used drugs commonly associated with Chemsex by aggregating reported use of poppers and/or cocaine and/or amphetamines and/or GHB/GBL and/or synthetic cathinones at home or someone else's home. Two categorical variables were then created for both general drug use and Chemsex, coded as non-other drug user, users of a specific drug (e.g. Poppers), and non-user of said drug but user of another drug to “feel better or get high".

### Other variables

Demographic and epidemiological variables included participants’ age, sex (male or female), HIV transmission risk group (MSM, heterosexual contact, IV drug use or other), years since diagnosis (0–10, 11–20; 21–30, > 30 years), and the viral load measure closest to the QuAliV assessment (± 2 years). We defined viral suppression as a measure of < 50 copies/mL.

### Statistical analysis

We used univariable and multivariable linear regression models to study the mean difference in CD4/CD8 ratios between users of each drug and non-users. We adjusted all analyses for age, sex, HIV risk group, time since diagnosis and other drug use, which were considered as potential confounders a priori. We equated a p-value < 0.05 with statistically significance. Missing values for drug use were encountered and reasons for missingness investigated. Study participants who did not answer all items differed from those who did. They were older or more likely to be female depending on the drug. Since these groups were more likely to be unfamiliar with the drugs in question, we considered that missing data likely depended on the intensity of drug use and were therefore “Missing Not At Random”. We therefore performed a complete case analysis. All statistical analyses were performed in R version 3.6.1 and R studio version 1.0.153.

## Results

Of the 929 participants who had completed the self-assessment prior to 31 December 2019, 867 (93.3%) had completed the "My lifestyle" module. One-hundred and thirty-four participants who lacked CD4 and/or CD8 cell count measure, 44 participants who were not virally-suppressed (last viral load ≥ 50 copies/mL) and 29 who lacked a recent last viral load measure and were excluded. We ultimately considered 660 participants.

As described in Table 1, participants’ mean age was 54.7 (standard deviation [s.d.] 11.2) years old and 72.4% [487/660] were male of whom 65.3% were MSM [318/487]. Twenty-two percent [147/660] had been diagnosed with HIV for 0–10 years, 25% [170/660] for 11–20 years, 36% [243/660] for 21–30 years and 15% [100/660] for more than 30 years. Their mean CD4/CD8 ratio was 1.1 (s.d. 0.6) and 47.7% [315/660] had a CD4/CD8 ratio of < 1. Thirty-five percent [225/660] were current smokers and 34.5% [225/660] had a history of smoking. Eighty-three percent [539/660] reported being current drinkers, among whom hazardous drinking and alcohol use disorder were detected in 36.8% [185/539] and 2.2% [11/539] respectively. Approximately twenty percent [129/660] used cannabis and nearly twelve percent [76/660] used other drugs, the most common of which were poppers (82.9%, n = 63), synthetic cathinones (50.0%, n = 38), and cocaine (34.2%, n = 26). We considered 90.8% [69/76] of those who reported other drug use to be using drugs commonly associated with Chemsex based on the aforementioned criteria.

**Table 1:** Demographic, clinical and substance use in virally suppressed people living with HIV, QuAliV -ANRS CO3 AQUIVIH-NA Cohort, 2018–2019, Nouvelle-Aquitaine, France) (N = 660)

	Statistics*		
Demographics			
Age (mean, s.d.)	54.7 ± 11.2		
Sex (% male)	487 (72.4)		
Country of birth (% born in France)	561 (85.0)		
HIV infection			
Time since diagnosis (years) (mean, s.d.)	20.0 ± 9.7		
HIV transmission risk group			
Men who have sex with men	318 (48.2)		
IV drug use	70 (10.6)		
Heterosexual contact	229 (34.7)		
Other	43 (6.5)		
Last CD4 count (cells/mm^3^)	736.4 ± 333.8		
Last CD8 count (cells/mm^3^)	775.1 ± 387.5		
Last CD4/CD8 ratio	1.1 ± 0.6		
	1.0 [0.0–5.7]		
< 1	315 (47.7)		
≥ 1	345 (52.3)		
Drug use			
Tobacco			
Current smoker	225 (34.5)	Very low to low nicotine addiction^1^	52 (31.3)
		Medium nicotine addiction^1^	55 (33.1)
		Strong nicotine addiction^1^	37 (22.3)
		Very strong nicotine addiction^1^	22 (13.3)
Former smoker	225 (34.5)		
Never smoker	202 (31.0)		
Alcohol			
Current drinker	539 (82.8)	No misuse^2^	307 (61.0)
		Hazardous drinking^2^	185 (36.8)
		Alcohol use disorder^2^	11 (2.2)
Non-drinker	112 (17.2)		
Cannabis			
Current user (last 12 months)	129 (19.9)		
Non-user	519 (80.1)		
Other drugs			
Current user (last 12 months)	76 (11.9)		
Non-user	563 (88.1)		
		Use in a private home	
Poppers	63 (82.9)		53 (84.1)
Cocaine	26 (34.2)		24 (92.3)
Amphetamines/ecstasy/MDMA/methamphetamines	21 (27.6)		9 (42.9)
Synthetic cathinones	38 (50.0)		37 (97.4)
GHB GBL	19 (25.0)		18 (94.7)
Opiates	3 (3.9)		3 (100.0)
Synthetic cannabinoids	0 (0.0)		-
“Chemsex drug user”**	69 (90.8)		

*Data are presented as n (%), mean ± SD, or median [min–max]

^1^based on the Fagerström test score, ^2^based on the Audit-C test score

**Poppers and/or cocaine and/or amphetamines and/or synthetic catinones and/or GHB/GBL used in a private home

We present the results of univariable and multivariable analyses conducted for each drug in Table 2. Mean CD4/CD8 ratios of participants using tobacco, alcohol and cannabis was not found to be significantly different compared to non-users in neither univariable nor multivariable analyses. The CD4/CD8 ratio of participants using at least one Chemsex-associated drug was on average 0.226 (95% confidence interval [CI] − 0.383, − 0.070) lower than that of non-users in univariable analysis (p = 0.005) and 0.165 lower [95% C.I − 0.343, − 0.012] in multivariable analysis, however, the observed difference was only of borderline statistical significance (p = 0.068). In multivariable analyses, considering each drug individually, we failed to find statistically significant mean differences in CD4/CD8 ratios in users compared to non-users.

**Table 2:** Univariable and multivariable analyses of smoking, alcohol, cannabis, and drug use compared to non-use in PLWH, QuAliV-ANRS CO3 AQUIVIH-NA Cohort, 2018–2019, Nouvelle-Aquitaine, France (N = 660)

	Univariate analysis				Multivariate analysis*		
	Non-user CD4/CD8 ratio (β0)	Mean CD4/CD8 ratio difference	95% interval confidence	p-value^#^	Mean CD4/CD8 ratio difference	95% interval confidence	p-value^#^
Tobacco^1^	1.12						**0.391**
Non-daily smoking or very low to low dependence vs. non-smoking^a^		− 0.013	− 0.152; 0.127	0.857	0.014	− 0.136; 0.165	0.851
Medium dependence vs. non-smoking^b^		0.087	− 0.090; 0.264	0.333	0.161	− 0.030; 0.351	0.098
High to very high dependence vs. non-smoking		− 0.068	− 0.239; 0.104	0.439	− 0.016	− 0.199; 0.167	0.863
Alcohol^2^	1.10						**0.371**
No misuse versus non-drinking		0.001	− 0.135; 0.138	0.986	0.054	− 0.089; 0.197	0.460
Misuse or dependence versus non-drinking		0.062	− 0.085; 0.209	0.406	0.108	− 0.045; 0.260	0.167
Cannabis	1.13						
Current user vs. non-user		− 0.014	− 0.135; 0.107	0.819	0.027	− 0.124; 0.179	0.723
Poppers	1.15						
Current user vs. non-user		− 0.222	− 0.385; − 0.059	0.008	− 0.159	− 0.341; 0.023	0.086
Cocaine	1.15						
Current user vs. non-user		− 0.142	− 0.388; 0.105	0.259	− 0.086	− 0.371; 0.200	0.557
Amphetamines/ecstasy/MDMA/methamphetamines	1.15						
Current user vs. non-user		− 0.010	− 0.283; 0.262	0.941	0.092	− 0.223; 0.406	0.567
Synthetic cathinones	1.15						
Current user vs non-user		− 0.216	− 0.422; − 0.010	0.039	− 0.167	− 0.400; 0.065	0.157
GHB/GBL	1.15						
Current user vs. non-user		− 0.229	− 0.515; 0.057	0.118	− 0.180	− 0.483; 0.122	0.242
« Chemsex drug user»^3^	1.15						
Current user vs non-user		− 0.226	− 0.383; − 0.070	0.005	− 0.165	− 0.343; 0.012	0.068

*Adjustment factors: age, sex, HIV transmission risk group, time since diagnosis, other substance use

^#^T-test or Fisher test (bold)

^1^Based on the Fagerström test score, ^2^Based on the Audit-C test score, ^3^Poppers and/or cocaine and/or amphetamines and/or synthetic catinones and/or GHB/GBL use in a private home

^a^“The average CD4/CD8 ratio is 0.013 lower in non-daily smokers or smokers with a very low to low nicotine addiction compared to non-smokers.

^b^“The average CD4/CD8 ratio is 0.087 higher in smokers with a medium nicotine addiction compared to  non-smokers

## Discussion

Although we did not find a statistically significant mean difference in CD4/CD8 ratio between users and non-users of a specific drug, our analysis suggests that drug use in the context of Chemsex may be associated with a lower CD4/CD8 ratio. Indeed, univariable analyses showed a lower CD4/CD8 ratio among users of Poppers and synthetic cathinones, drugs which are very commonly used for Chemsex as well as among users of at least one Chemsex associated drug (poppers and/or cocaine and/or amphetamines and/or GHB/GBL and/or synthetic cathinones) at home or someone else's home. These associations were statistically significant or close to significant. Multivariable analyses followed the same trend, suggesting a lack of statistical power in our analyses. These results therefore do not seem to incriminate a particular drug but rather the context of use and thus a group of at-risk users in whom certain characteristics or lifestyle factors appear to hinder immune restoration in spite of viral suppression. A longitudinal study conducted in the United States found no association between polydrug use and lymphocytes in PLWH, however, this study was conducted in pre HAART era, before the emergence of drug use trends like Chemsex, making comparisons futile [19].

Since Chemsex has increased in the last ten years, the lower CD4/CD8 ratio that we found in Chemsex-associated drug user compared to non-users could have been due to a recent diagnosis and initiation of ART. However, those considered as using Chemsex-associated drugs had been in care and receiving ART for more than 10 years in addition to being virally suppressed. Furthermore, compared to the overall study population, these participants had higher mean CD4 and CD8 counts, which supports the chronic inflammation hypothesis. A recent study comparing MSM and heterosexual men, all of whom were HIV-negative, found MSM and MSM reporting multiple recent partners more specifically to have lower CD4/CD8 ratios on average. Cytomegalovirus (CMV) infection was hypothesized to contribute to observed differences, but drug use did not appear to be associated [24]. Unfortunately, we were not able to account for CMV infection in our analysis due to data reliability concerns.

We found no association between tobacco use and a low CD4/CD8 ratio. However, smoking has been associated with disturbances in lymphocyte function, specifically an increase in lymphocyte activation and an increase in helper CD4 T-lymphocytes and thus an increase in the CD4/CD8 ratio [14, 25, 26]. Our results also show no association between alcohol use and the CD4/CD8 ratio. Several studies have shown that PLWH who are heavy drinkers tend to have lower CD4 cell counts than more moderate drinkers, which seems to contradict our findings [27, 28], yet other studies have not shown alcohol consumption to be associated with CD4 cell count levels in PLWH [29]. A recent longitudinal study found that exclusive cannabis use had no effect on PLWH’s lymphocytes but that other drug use was associated with increased CD8 cell activation [20]. These results are consistent with ours.

While our study provides quality data on PLWH’s recent drug use patterns and raises questions for future studies, we are also aware of its limitations. It was conducted in a non-random sample of those in care in Nouvelle Aquitaine and therefore might not be generalizable to all of those in care in our region, namely non-users who might be less likely to participate. Nevertheless, MSM, who are the most likely to engage in Chemsex, appear to be well-represented. Furthermore, there is strong evidence that data on sensitive topics like drugs use can be collected reliably using anonymous validated instruments as these methods are less subject to social desirability bias. Our study is cross-sectional and therefore does not account for drug use and CD4/CD8 ratio trajectories over the course of HIV infection and its treatment. We assessed current drug use within the past 12 months, which may or not reflect participants’ drug use history. It is therefore possible those in the non-user group previously engaged in drug use. This type of misclassification would have resulted in a dilution of effect. This might be one explanation of non-significant findings, namely those which are discordant with previous research. Finally, we relied on CD4 and CD8 cell count measures recorded within 2 years of the self-reported assessment. While this is an ostensibly large window in which to draw on participants’ available laboratory data, we feel that it is acceptable given our study sample’s characteristics, the exclusion of those who were not completely virally-suppressed, and CD4 and CD8 cell counts trajectories over the course of HIV treatment. Finally, the relatively small number of drug users in our sample may have resulted in low statistical power.

## Conclusions

While we did not find an association between tobacco, alcohol and cannabis use and the CD4/CD8 ratio, our results suggest that those who use Chemsex-associated drugs could represent a population at risk of chronic inflammation. The specific determinants of which merit further investigation.

## Data Availability

The anonymized individual data and the data dictionary of the study will be made available to other researchers by the coordinating investigator, Professor Fabrice Bonnet (fabrice.bonnet@chu-bordeaux.fr) after approval of a methodologically sound proposal by the study's steering committee and the signature of a data access agreement.

## Abbreviations

ART: Antiretroviral therapy
CD4/CD8 ratio: CD4 to CD8 T cell count ratio
CI: Confidence interval
CMV: Cytomegalovirus
HIV: Human Immunodeficiency Virus
MSM: Men who have sex with men
PRO: Patient-reported outcomes
PLWH: People living with HIV
s.d.: Standard deviation
